# Dissociation of neural correlates of verbal and non-verbal visual working memory with different delays

**DOI:** 10.1186/1744-9081-3-56

**Published:** 2007-10-25

**Authors:** Christoph Rothmayr, Oliver Baumann, Tor Endestad, Roland M Rutschmann, Svein Magnussen, Mark W Greenlee

**Affiliations:** 1 Institute of Experimental Psychology, University of Regensburg, Germany; 2 Center for the Study of Human Cognition, Department of Psychology, University of Oslo, Norway; 3 Department of Psychology, University of Glasgow, UK

## Abstract

**Background:**

Dorsolateral prefrontal cortex (DLPFC), posterior parietal cortex, and regions in the occipital cortex have been identified as neural sites for visual working memory (*WM*). The exact involvement of the DLPFC in verbal and non-verbal working memory processes, and how these processes depend on the time-span for retention, remains disputed.

**Methods:**

We used functional MRI to explore the neural correlates of the delayed discrimination of Gabor stimuli differing in orientation. Twelve subjects were instructed to code the relative orientation either verbally or non-verbally with memory delays of short (2 s) or long (8 s) duration.

**Results:**

Blood-oxygen level dependent (BOLD) 3-Tesla fMRI revealed significantly more activity for the short verbal condition compared to the short non-verbal condition in bilateral superior temporal gyrus, insula and supramarginal gyrus. Activity in the long verbal condition was greater than in the long non-verbal condition in left language-associated areas (STG) and bilateral posterior parietal areas, including precuneus. Interestingly, right DLPFC and bilateral superior frontal gyrus was more active in the non-verbal long delay condition than in the long verbal condition.

**Conclusion:**

The results point to a dissociation between the cortical sites involved in verbal and non-verbal *WM *for long and short delays. Right DLPFC seems to be engaged in non-verbal *WM *tasks especially for long delays. Furthermore, the results indicate that even slightly different memory maintenance intervals engage largely differing networks and that this novel finding may explain differing results in previous verbal/non-verbal *WM *studies.

## Background

*Working Memory *(*WM*) is the ability to keep a limited amount of information online for immediate use during short intervals [1]. In typical *WM *experiments 1 to 10 items are maintained in memory for periods up to and including 60 s [2]. The classical model of *WM *consists of the *central executive *and three subsidiary systems, namely the *visuo-spatial sketchpad*, the *phonological loop*, and the recently proposed *episodic buffer *[3].

A memory system related to the *visuo-spatial sketchpad *component of *WM *is *perceptual memory*, which has been described as a low-level memory process that is comprised of a series of independent parallel mechanisms for various basic stimulus dimensions. These attributes, such as spatial frequency, contrast, or orientation, are thought to be the building blocks of visual images [4]. According to this theory, each attribute is stored with high precision in separate perceptual stores [5]. These models of sensory-based WM emphasize the delay-related signals in sensory cortex and the reciprocal projections of these areas to parietal and prefrontal cortex [6].

Objects in visual *WM *may be encoded with the help of verbal or non-verbal strategies. Numerous studies have investigated verbal and non-verbal WM [7-9]. The stimuli to test verbal and non-verbal WM differ significantly, ranging from single letters, numbers, dots, squares to complex objects and scenes [10-12]. The extent to which these stimuli can be coded verbally represents a major confound in these studies [13], since the labels given by the observer to the material, and not their visual representations *per se*, will be stored.

Several brain areas have been identified as the neural correlates of visual *WM *by means of lesion studies [14-16], PET [17,18], ERPs [19], and fMRI [20,21]. Among these is posterior parietal cortex, which may reflect the neural capacity limit of visual *WM *[22]. Recently, Xu and Chun [23] have proposed that the inferior intraparietal sulcus (IPS), the superior IPS, and lateral occipital cortex (LOC) work in parallel to support visual *WM *encoding and maintenance. They suggest that representations in inferior IPS may be limited to a fixed number of objects, whereas capacity in LOC and superior IPS is limited by object complexity. LOC and superior IPS may thus participate in storing detailed representations of stimuli in visual *WM*. In addition, various striate and extra-striate areas of the occipital cortex have been identified as visual *WM *correlates [24]. Interestingly, relatively early visual areas beyond V1, which have previously only been associated with visual perception, are also active during visual *WM *delays [6]. Virtually all studies that investigated visual *WM *found activity in prefrontal cortex (PFC). The dorsolateral prefrontal cortex (DLPFC; BA 46/9) seems to play a crucial role in *WM*-related processes [25-27]. DLPFC activity has foremost been found in studies that required the manipulation of relevant items in memory [20,28]. Most of these studies have used *n*-back tasks in which the subject has to remember an item presented *n*-trials ago and match it to the present item. Delayed-discrimination tasks, on the other hand, show less DLPFC activity [29]. During delayed-discrimination tasks an item has to be discriminated from the previously presented item. Thus, the mere maintenance of an item and not its manipulation is required. Several review articles point to a role for DLPFC in the active manipulation of material in visual WM [7-9]. However, other evidence suggests that DLPFC is involved in the storage of visual information for several objects [30].

Further studies have attempted to identify brain regions related to either verbal or non-verbal *WM*. Based on the identified neural networks a verbal/non-verbal dissociation has been suggested in either a ventral/dorsal or a left/right fashion [2,31]. Using a 2-back task, Ikeda and Osaka [17] investigated memory for colours that could be coded either verbally or visually. Analysis of the results from the condition where colours could be coded verbally revealed activity in areas associated with the *phonological loop*, such as inferior frontal gyrus and inferior parietal lobule. The non-verbal coding of colours resulted in right inferior frontal gyrus activity, an area that has been associated with the *visuo-spatial sketchpad *of *WM*. These results stand in contrast to the results of the review article by Cabeza and Nyberg [7] of more than 60 visual *WM *studies. These authors concluded that there is little evidence for a dissociation of verbal and non-verbal *WM *in the human cortex. This could be explained by the observation that most paradigms allow for the verbal encoding of visual material. Although Ikeda and Osaka [32] revealed a possible dissociation between verbal and non-verbal *WM*-associated brain areas, their non-verbal stimuli may also have been coded verbally by the subjects. The words "lighter" or "darker" may have been used by the subjects for the intended non-verbal stimuli that all stemmed from one color category. Although their study revealed differing brain activity between verbal and non-verbal conditions this does not imply that this was due to their subjects' coding approaches. Mere differences in the visual appearance of the stimuli could also, at least in part, account for their results.

The effect of memory delay length on cortical activation has received less attention. In the visual *WM *studies reviewed above, inter-stimulus intervals (ISIs) in delayed discrimination paradigms varied from 350 ms [29] to 24 s [30]. Barch et al. [33] reported on the impact of delay length on brain activation in visual *WM *tasks. In their verbal *WM *task the retention interval was either 1 or 8 s. The task used was a variant of the Continuous Performance Test [34]. Subjects had to press a button whenever the letter X followed the letter A. The fMRI data revealed increased activation for the longer delay in inferior frontal gyrus, left posterior parietal lobe, and the left DLPFC. The previously mentioned conflicting results with respect to a dissociation of verbal/non-verbal *WM *may be, in part, due to the varying retention intervals used [33]. Differences in task demands ranging from simple delayed-discrimination to demanding n-back tasks may also underlie the differences in brain activation.

The present study attempts to account for some of the inconsistencies in visual *WM *studies by systematically varying both delay length and coding strategies in the discrimination of simple grating stimuli. We used Gabor stimuli of differing orientation and instructed subjects to explicitly encode the relative orientations using a verbal code. The results from this condition were compared to those arising from a condition, where verbal coding could not be readily employed. We believe that we were able to create a paradigm in which non-verbal stimuli were virtually identical to the verbal stimuli but which could not be coded verbally as may have taken place in previous *WM *studies. Our findings suggest that the coding strategy used by the subjects has a profound effect on the pattern of brain activation exhibited during the delayed discrimination of similar stimuli. These differences are most pronounced for the long delay, where verbal stimuli seem to engage predominantly left-hemispheric temporo-parietal areas, whereas non-verbal memory is associated with medial and right-hemispheric frontal brain activity.

## Materials and methods

### Subjects

Twelve right-handed adults (6 male, 6 female), aged between 20 and 40 years (mean= 25.4 yrs), participated in the study. All participants gave their written informed consent. All had normal or corrected-to-normal vision and reported no prior psychiatric or neurological impairments.

### Task

In the experiment the participants had to decide whether two Gabor stimuli, which were presented sequentially and separated by a delay period, had the same or a different orientation. The inter-stimulus interval (ISI) between the reference and the test stimulus was either 2 or 8 seconds. Gabor pairs were constructed so that they could be coded either verbally or non-verbally. Thus, the experiment consisted of four conditions (verbal/non-verbal x ISI 2 s/ISI 8 s).

**Figure 1:**
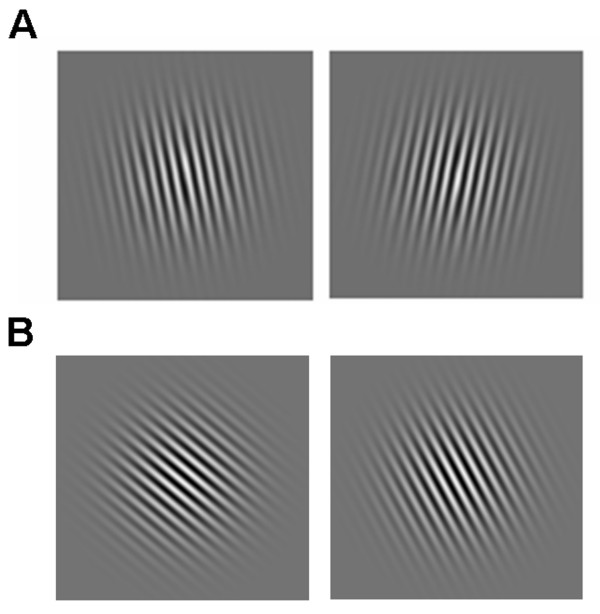
A. An example of a reference Gabor stimulus with its corresponding test stimulus for the verbal condition, in which the participants were instructed to memorize the orientation with a sub-vocal verbal rehearsal strategy (e.g. "left", "right" of vertical). The example depicts stimuli on a trial in which the test and the reference grating differed. B. An example of a stimulus pair for the nonverbal conditions, in which the instructions emphasized the use of visual encoding. Here the stimuli are taken from a trial in which the test and the reference stimulus differed.

In the verbal conditions stimuli were either oriented to the left (79°C) or to the right (101°C) of vertical, resulting in a difference in angle of 22°C. This was done so that subjects could verbally code these orientations with the words "left" and "right", as it had been suggested to them in the instruction. An example stimulus pair from the verbal conditions is depicted in Figure 1A for a "different" trial. In the two non-verbal conditions three reference Gabors were used that were oriented at either 34°C, 40°C, or 46°C, with respect to horizontal (0°C). Corresponding test stimuli had an orientation that was 22°C greater or lesser than that of the reference stimulus, or it had the same orientation. Gabors were constructed in this manner so that they could not be easily coded in a verbal manner (i.e., reference to the principal axes did not ease the task) but demanded perceptual coding. An exemplary non-verbal stimulus pair is shown in Figure 1B for a trial in which the reference and the test grating differed.

In 50% of all trials both the reference and the test stimulus had the same orientation, on the other trials the reference and test stimuli differed in orientation. Trials were presented in random order and subjects were instructed to maintain central fixation throughout the experiment.

**Figure 2:**
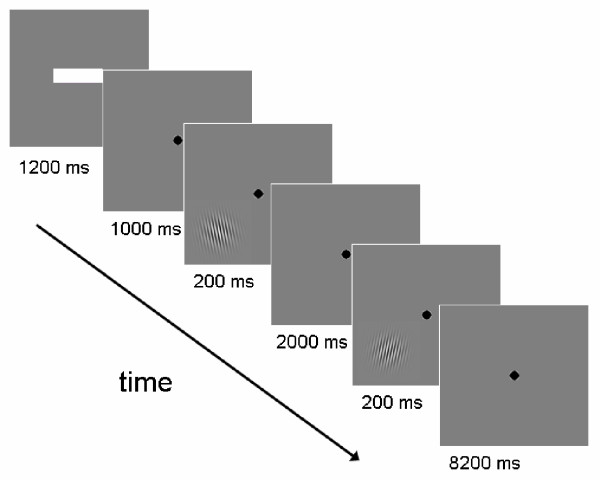
Schematic depiction of a trial from the verbal ISI 2s condition in which the test and the reference stimulus differed. Trials started with a bar that informed subjects about delay length (short bar: 2 s, long bar: 8 s) and type of stimulus pair (green bar: verbal, red bar: non-verbal). After this a fixation point appeared that remained in the centre of the screen for the rest of the trial. This was followed by the reference Gabor that was shown in either the upper right or lower left quadrant of the screen (here a trial with stimuli in the lower left quadrant are presented). The delay interval was presented afterwards (2 or 8 s), followed by the test Gabor that appeared in the same quadrant as the previous reference Gabor. During the following interval, the subject had to judge if the test and the reference stimulus had the same or a different orientation and press the corresponding button.

At the beginning of each trial, a red or green bar appeared for 1000 ms in the centre of fixation. A red bar signified that a non-verbal stimulus pair was coming up, while a green bar stood for a verbally codable stimulus pair. The bar was either short or long. A short bar indicated an upcoming short ISI (2 s) and a long bar indicated a long ISI (8 s). Subjects were cued in this way on each trial to optimize their respective coding strategies. This cue was followed for 1200 ms by a black fixation point in the centre of the screen. Then the reference grating appeared for 200 ms in either the lower left or the upper right quadrant of the screen, with the fixation point still remaining in the centre of the screen. Gabors were presented in the periphery (see below). During the following ISI (either 2000 or 8000 ms), only the fixation point appeared on the screen. After this the test grating appeared in the same quadrant as the reference Gabor for 200 ms. Subjects then had to press a button with the index finger of their right hand if they thought that the test and the reference grating had the same orientation. Another button was pressed with the middle finger of the right hand if they thought that the two orientations differed. Participants had been instructed to respond as quickly and as accurately as possible. After the offset of the test Gabor, a fixation point appeared for either 8200 ms (for the 2s ISI) or for 2200 ms (for the 8s ISI). A schematic depiction of a trial for the short retention interval (ISI 2s, verbal), in which the reference and the test stimulus were the same, is depicted in Figure 2.

Prior to the fMRI experiment subjects participated in a training session outside the scanner (n = 40 trials). In the fMRI experiment, each subject participated in one session that consisted of a total of 144 trials. At the end of the session, subjects were asked if and how often they had used verbal coding strategies in both the verbal and the non-verbal conditions.

### Display and stimulus parameters

Stimuli were created with Matlab 6.5.1 software (Math Works Inc., Natick, MA) and presented with Presentation 9.13 software (Neurobehavioral Systems Inc., Albany, CA). Stimuli were back-projected on a screen inside the scanner with a D-ILA LCD-projector (JVC Corp., Japan) with a frame refresh rate of 60 Hz. The screen size subtended 16.4°C × 21.7°C of visual angle. Gabor stimuli had a diameter of approximately 6.5°C of visual angle and were presented in the lower left quadrant or the upper right quadrant of the screen at a visual angle of 8.6°C from central fixation, measured from the centre of the Gabors. Gabor patches had a maximum contrast close to 100% and a spatial frequency of 3.4 c/deg. The contrast of the Gabors was tapered with a Gaussian kernel (Gauss constant: 1.3 deg).

Subjects responded by pressing the buttons of a Lumitouch (Photon Control, Burnaby, Canada) optical response device with their index finger and the middle finger of their right hand. Reaction time (RT) and accuracy data were recorded and stored for offline analysis.

### fMRI methods

Blood-oxygen-level-dependant imaging data were acquired with a 3-Tesla Siemens Allegra head scanner (Siemens Inc., Erlangen, Germany) at the University of Regensburg. The scanner acquired echo-planar-imaging (EPI) sequences using fast gradients. A standard one-channel head coil was used. During T2* image acquisition 34 slices (whole brain) were scanned in interleaved order. Time-to-repeat (TR) was 2000 ms. Time-to-echo (TE) was set at 30 ms, with a flip angle of 90°C. Voxel-size was set to 3 × 3 × 3 mm. The field of view measured 192 × 192 mm. Trials in the experimental paradigm were synchronized with scanner pulses. In every experimental session, 1088 scans were acquired. In order to obtain a better estimate of the actual hemodynamic response function (hrf) a jitter was implemented during the acquisition of functional images. Therefore on half of the trials in the experimental paradigm the trial onset was shifted by a fixed amount of time. A 1000 ms fixation period was added at the beginning and at the end of each respective trial, thus shifting events in the jittered trials by 1000 ms. Anatomical T1-weighted images were obtained using a MPRAGE pulse sequence (Magnetization Prepared RApid Gradient Echo) with time-to-repeat (TR) of 2300 ms, a time-to-echo (TE) of 3.93 ms, and a flip angle of 12°C. A total of 176 slices were scanned, with isotropic voxels sized 1 × 1 × 1 mm. The field of view had a size of 256 × 256 mm.

### Data analysis

Reaction time and accuracy data were analyzed statistically with SPSS for Windows 12.0 software (SPSS Inc., Chicago, IL). A repeated-measures ANOVA was conducted at a significance level of p ≤ 0.05.

Images were pre-processed and statistically analyzed with SPM2 [35] which runs in MatLab (Math Works Inc., Natick, MA). Prior to pre-processing all obtained imaging data in DICOM format were transformed to ANALYZE file format. Functional data were slice timed and realigned. A T2*-weighted mean image of the unsmoothed images was co-registered with the corresponding anatomical T1-weighted image of the same individual. The individual T1-image was used to derive the transformation parameters for the stereotaxic space using the SPM2 template (Montreal Neurological Institute (MNI) Template), which was then applied to the individual single co-registered EPI images. The voxel sizes of the written normalised images were 1 mm^3^. Images were then smoothed with a 8-mm full-width half maximum (FWHM) isotropic Gaussian kernel.

Statistical evaluation consisted of modeling the onset times of the test Gabor-stimuli as events on individual first level. These onsets were modeled separately for each of the 4 conditions if the correct response was given. Another two regressors for incorrect responses after an ISI of 2 or 8 seconds, respectively, were also included amounting to a total of 7 regressors (including constant) for each individual analysis. Interesting effects were contrasted using T-statistics, generating the relevant contrast images for second level evaluation.

For the random-effects group level statistics, T-value maps were calculated with appropriate contrast images. Activation vs. baseline maps were thresholded at p < .05 corrected on cluster level (cluster-defining threshold t = 4.0). Thresholds were adjusted for differential contrasts as we expected only small differences of effect sizes. Clusters surpassing an individual threshold of p < .05 corrected on cluster level (cluster-defining threshold t = 2.0) are reported as significant differential activations. To visualize the results, the activations were overlaid on a normalized rendered image from one of the subjects.

## Results

### Behavioural results

The computation of each individual's performance revealed that all participants were able to discriminate the relevant stimuli reasonably well. Mean accuracy (proportion of correct responses) for the four conditions was as follows: verbal, 2s ISI: 0.958 (standard error of the mean, SE = 0.013); verbal, 8s ISI: 0.949 (SE = 0.017); non-verbal, 2s ISI: 0.775 (SE = 0.024) and non-verbal, 8s ISI: 0.778 (SE = 0.022). A repeated-measures ANOVA with the factors type of stimulus (verbal/non-verbal) and ISI (2 s/8 s) revealed a significant effect of type of stimulus [F(1,11) = 55.27, p ≤ 0.01]. Accuracy was correspondingly higher for the verbal conditions.

**Figure 3:**
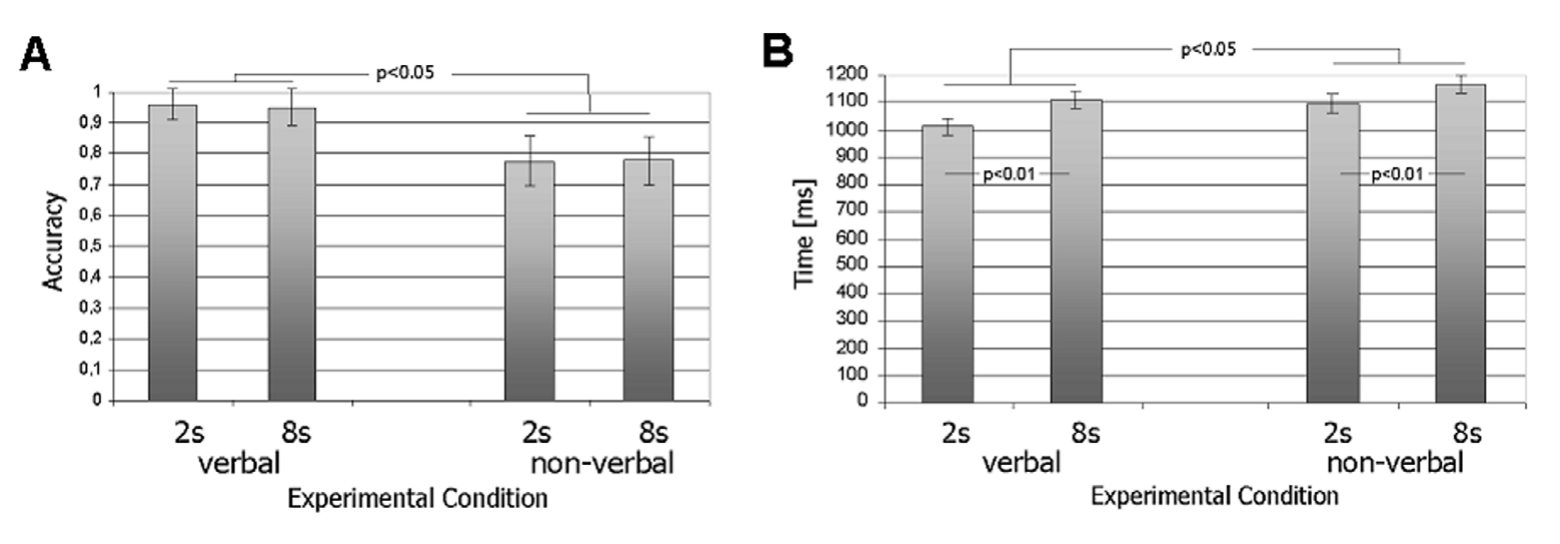
*A*. Mean reaction times are presented for the non-verbal and the verbal conditions. Reaction times in the non-verbal trials were significantly higher than in the verbal conditions [F(1,11) = 8.27, p ≤ 0.05]. There was a significant increase in the reaction time for the 8s ISI when compared to the 2s ISI [F(1,11) = 29.19, p ≤ 0.01]. *B*. Performance (portion correct responses) in the verbal and the non-verbal 2 and 8s ISI conditions. Accuracy in the verbal conditions was higher than in the non-verbal conditions [F(1,11) = 55.27, p ≤ 0.01].

Reaction times (RTs) were computed for correct trials only and were as follows: verbal, 2s ISI: 1012 ms (SE = 30 ms); verbal, 8s ISI: 1109 ms (SE = 31 ms); non-verbal, 2 s ISI: 1096 ms (SE = 35 ms); non-verbal, 8s ISI: 1168 ms (SE = 30 ms). An ANOVA revealed a significant main effect for the factor type of stimulus [F(1,11) = 8.27, p ≤ 0.05] and a highly significant main effect for the factor ISI [F(1,11) = 29.19, p ≤ 0.01]. Thus, RTs in the verbal conditions were significantly lower than in the non-verbal conditions. Also, RTs in the long retention (8 s) conditions were significantly longer when compared to the short retention (2 s) conditions, in agreement with earlier psychophysical results [36]. The portion of correct responses and RTs for all four conditions (averaged over all participants) are depicted in Figure 3.

The examination of the responses given by the subjects after having been asked about their coding strategies revealed that the overwhelming majority of them used verbal coding for all verbal trials (92% of subjects) and refrained from doing so in the non-verbal trials (75% claimed to have used verbal coding at no time or only seldom). The few subjects who had attempted to use verbal coding for non-verbal trials reported to have used the words "tilted"/"more tilted". These subjects also claimed to have aborted the strategy soon after the onset of the experiment because they had felt that it was not successful. The different results for verbal versus non-verbal trials may therefore be regarded as a consequence of the participants' coding strategies. All participants claimed to have used the words "left" and "right" of vertical for the verbal coding trials in covert speech.

### Functional imaging results

Results from the contrasts against baseline are displayed in Table 1. The hemisphere, anatomical region, corresponding Brodmann area number, the MNI location, as well as the magnitude and size of the activated cluster are given for each of the four conditions. The patterns of activation indicate that the brain activity resulting from the verbal and non-verbal conditions are widely spread across prefrontal, cingulate, parietal, temporal and occipital regions in both hemispheres.

**Table 1:** Brain areas showing significant activation. Contrasts: verbal 2s ISI > baseline, verbal 8s ISI > baseline, non-verbal 2s ISI > baseline, and non-verbal 8s ISI > baseline. The Montreal Neurological Institute (MNI) coordinates of the most active voxel is given for each cluster, along with the z-value of the magnitude of activation and the number of voxels contained within the cluster (in parentheses). Abbreviations for each brain structure assigned using the SPM2 extension MSU: MFG = middle frontal gyrus; IFG = inferior frontal gyrus; IPL = inferior parietal lobule; STG = superior temporal gyrus; SFG = superior frontal gyrus; MTG = middle temporal gyrus

			**MNI coordinates**			
***Hemisphere *& Region**	**Brodmann Area**	**Hemisphere**	**x**	**y**	**z**	**Z-values of maxima (cluster size in number of voxels)**

**Verbal 2s ISI > baseline**						

cingulate gyrus, IPL, MFG, postcentral gyrus, precentral gyrus	4/6/24/44	L	-2	8	52	5.92 (13387)
cerebellum	N/A	L/R	8	-54	-10	5.61 (2642)
cuneus, posterior cingulate, precuneus	30/31	L/R	24	-44	0	5.18 (1464)
IPL, postcentral gyrus, precuneus	2/7/40	R	20	-64	46	5.15 (1883)
Insula, MFG	6/13/44	R	30	50	30	4.82 (1626)

**Verbal 8s ISI > baseline**						

cingulate gyrus, insula, precentral gyrus	4/6/24/44	L/R	6	-26	0	5.88 (22831)
MFG	6	R	30	-8	60	4.79 (184)
postcentral gyrus, precentral gyrus	9/10	R	32	58	22	4.59 (248)
MFG, SFG	9/10	L	-42	26	30	4.57 (371)
MTG, STG	39	R	50	-58	6	4.15 (113)
MFG	6	R	36	-2	38	4.13 (275)
MFG, SFG	9/10	R	32	58	22	3.92 (272)

**Non-verbal 2s ISI > baseline**						

cerebellum	N/A	L/R	8	-60	-10	5.56 (2308)
IPL, postcentral gyrus	2/3/4/40	L	-38	-40	52	4.75 (1777)
IPL	2/40	R	38	-56	52	4.58 (1353)
IFG, insula	13/44	L	-48	8	16	4.58 (839)
cingulate gyrus, MFG	8/24/32	L/R	4	16	52	4.41 (795)
IFG, insula	13/47	R	40	16	2	4.35 (181)
cingulate gyrus	23	L/R	8	-32	32	4.32 (149)

**Non-verbal 8s ISI > baseline**						

IFG, insula, thalamus	9/13/41/44	L	-6	-18	4	5.85 (5935)
IFG, insula, IPL, MFG, precentral gyrus	13/23/30/40	R	40	16	0	5.84 (11186)
cingulate gyrus medial, frontal gyrus	23/24/33	L/R	4	18	48	5.82 (8829)
cuneus, precuneus	19	L	-30	-80	34	4.60 (339)

For our purposes, we focus on the comparison of activation across the different experimental conditions. The results for these differential contrasts (condition A > condition B) are displayed in Table 2. No significant activity was found for the contrast in which the activity arising in the non-verbal ISI 2s > verbal ISI 2s condition was compared. This lack of difference could be related to the temporal overlap of the BOLD response to the perceptual encoding and retrieval events in the non-verbal condition.

**Table 2:** Brain areas showing significant activation. Contrasts: verbal 2s ISI > non-verbal 2s ISI, verbal 8s ISI > non-verbal 8s ISI, and non-verbal 8s ISI > verbal 8s ISI, otherwise as in Table 1. No activity was detected in the contrast non-verbal 2s ISI > verbal 2s ISI. For abbreviations see Table 1

			**MNI coordinates**			
***Hemisphere *& Region**	**Brodmann Area**	**Hemisphere**	**x**	**y**	**z**	**Z-values of maxima (cluster size in number of voxels)**

**Verbal 2s ISI > non-verbal 2s ISI**						

insula, IPL, STG	13/41/42/43/44	R	52	-32	14	4.90 (2641)
insula, STG, supramarginal gyrus	13/40/41/42	L	-12	-12	20	3.91 (7176)
cingulate gyrus, posterior cingulate, precentral lobule	5/24/30/31	L/R	20	-48	-4	3.88 (6931)

**Verbal 8s ISI > non-verbal 8s ISI**						

cuneus, posterior cingulate, precuneus	7/23/30/31	L/R	8	-60	52	4.38 (3948)
IPL, MTG, STG, supramarginal gyrus	39/40/41/44	L	-62	-40	28	4.29 (2327)

**Non-verbal 8s ISI > verbal 8s ISI**						

medial frontal gyrus, SFG	32/9/8	L/R	8	22	50	4.70 (2934)
IFG, MFG	8/9/46	R	54	8	24	3.24 (1004)

**Figure 4:**
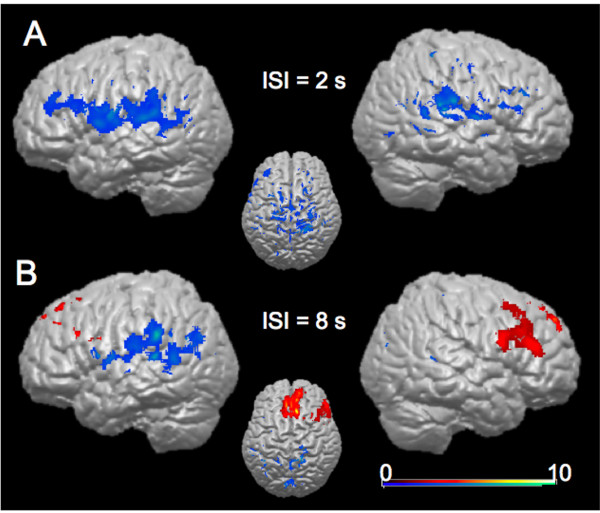
Results from the random-effects group-analysis. *A. B*rain areas showing significant activation in the contrast verbal 2s ISI > non-verbal 2s (blue shading). No significant activity was found for the contrast non-verbal ISI 2s > verbal ISI 2s. *B*. Significant activation in the conditions verbal 8s ISI > non-verbal (blue shading), and non-verbal 8s ISI > verbal 8s ISI (red shading).

Activity in the contrast verbal 2s ISI > non-verbal 2s ISI was detected in bilateral insula, superior temporal gyrus, and the right inferior parietal lobule. Significantly more BOLD-dependent activity was found in left SMG, posterior cingulate, right cingulate gyrus, and the right precentral lobule for this contrast. The contrast verbal 8s ISI > non-verbal 8s ISI revealed activity in the cuneus, posterior cingulate, middle temporal gyrus, superior temporal gyrus, and the inferior parietal lobule of the left hemisphere, as well as in the bilateral precuneus. The contrast non-verbal 8s ISI > verbal 8s ISI resulted in activity in bilateral superior frontal gyrus, left medial frontal gyrus, right inferior frontal gyrus, and right middle frontal gyrus. This differential activity, representing the mean differential contrasts for all participants, is depicted on a structural brain image of one of the subjects in Figure 4.

## Discussion

This study investigated differences in cortical BOLD activity for a verbal and non-verbal delayed-discrimination *WM *paradigm for short and long retention intervals. The paradigm used here, a delayed orientation discrimination task, focused on the maintenance of visual memory representations without any manipulation process. In the verbal encoding condition, Gabor patches were oriented slightly to the left or to the right of vertical so that subjects could covertly use the terms "left" and "right" as verbal cues. The "non-verbal stimuli" were oriented to the left only and could not be readily related to the vertical or horizontal axes. Gabors were constructed in this manner so that they could not be easily coded in a verbal manner (i.e., reference to the principal axes did not ease the task) but demanded perceptual coding. Differences in orientation angle between the reference and test gratings, however, were the same for both encoding conditions. We believe that subjects coded verbal and non-verbal stimulus pairs with a verbal coding strategy in one case and refrained from doing so in the latter instance. Firstly, subjects were explicitly told in the instruction to code verbal stimuli with the words "left" and "right".

Secondly, non-verbal stimuli were constructed in a fashion that would not lend themselves to verbal coding. Gratings in these conditions differed by 22°C (for "different" trials) and were all oriented to the left.

Orientations were selected that were not near prominent positions of an analogue clock face and stimuli were presented for 200 ms only. Verbal stimuli were oriented to the left or to the right of the vertical plane, thus inevitably yielding the verbal codes "left" and "right". Although usually considered an unreliable measure of experimental control, subject debriefings conducted in our experiment confirmed that subjects had used verbal coding in the verbal condition, and refrained from doing so in the nonverbal condition, as intended.

We believe that the stimuli used in this study represent a novel approach in the investigation of verbal and non-verbal *WM*. Due to the virtually identical visual appearance of the verbal and the non-verbal stimuli, differences in brain activity in this experiment can be attributed entirely to the coding strategies applied by the subjects. Indeed, the trial-by-trial cues instructed the subjects to apply the appropriate strategies to the individual trial types. This manipulation may not have been properly achieved in previous studies.

The systematic variation of delay length, as conducted here, presents a novelty in verbal/non-verbal *WM *research and may explain differing results as well.

The behavioural data revealed slower reaction times and lower accuracies for the non-verbal conditions as opposed to the verbal conditions, suggesting the use of different neural mechanisms.

Non-verbal *WM *is typically associated with the engagement of the visuospatial sketchpad component of *WM*, whereas verbal *WM *additionally engages the phonological loop component. It has frequently been reported in previous studies that verbal coding, as opposed to non-verbal *WM*, enhances *WM *performance, a finding that is reflected in this study's behavioural results.

Accuracies and reaction times differed between the verbal and non-verbal conditions (Fig. 3). It could be argued that we should have adapted the stimulus differences in angle between stimulus pairs or presentation time to yield equivalent performance for the two trial types. By doing this, however, differences in brain activity could not have been attributed to underlying coding strategies used by the subjects but would have to be explained in terms of differing visual stimulus properties. Such a procedure (i.e., different stimuli for verbal and nonverbal trial types), which was knowingly avoided in this study, may have constituted a major confound in previous studies. We believe that, although accuracies differed between verbal and non-verbal trials, the results may be interpreted as a result subjects' coding strategies and not to differing stimulus properties, a major problem in previous *WM *studies.

The functional imaging results presented here reflect maintenance processes dependent on both delay period and coding strategy applied. Since a simple delayed-discrimination *WM *paradigm was used here, it does not reflect manipulation processes that are usually captured in n-back tasks and that are thus hard to disentangle from maintenance processes [7-9].

The random-effects group analysis (Table 1), in which all four conditions were contrasted with baseline activation levels, revealed activity in prefrontal, posterior parietal cortex and further areas that have previously been associated with *WM*. The main focus of this study, however, was on the dissociation between verbal and non-verbal *WM *at different delay lengths. Therefore we will not discuss these results in detail, but rather focus on the direct comparisons of verbal and non-verbal conditions. The differential analysis between the verbal and non-verbal conditions revealed differing activity for the comparisons between the conditions with the same delay duration. In the short retention interval, significantly more activity was detected in bilateral areas close to well-known language areas, such as the supramarginal gyrus, superior temporal gyrus, and inferior frontal gyrus, with preponderance in the left hemisphere. No additional activity was found when contrasting the short non-verbal to the short verbal condition. In the long interval, however, the non-verbal condition showed more activity in right DLPFC and medial frontal areas than the verbal condition. In the verbal long-retention condition more activity could be measured in left language associated areas (such as supramarginal gyrus, superior temporal gyrus, as well as in medial parietal areas) when compared to that found in the long non-verbal condition.

These results suggest an interaction in visual *WM *between the effects of memory delay length and modality of encoding. The right DLPFC is significantly more active in the non-verbal condition with the long retention interval when compared to the verbal condition of same retention interval (Fig. 4). In contrast, in the long delay conditions, parietal, temporal, and frontal areas in the immediate proximity of language areas of the left hemisphere, as well as medial parietal areas, especially precuneus, were more active in the verbal than in the non-verbal condition. The neural basis for the *phonological loop *component of *WM *has been localized in left supramarginal gyrus, Broca's area, inferior frontal gyrus, and the superior parietal lobule [18,32]. Our study revealed relatively more activity in these same areas for the verbal coding condition and may thus indicate the engagement of the *phonological loop *for these conditions. On the other hand, the precuneus is a structure that has frequently been reported in connection with different forms of higher-order cognition including episodic memory retrieval [37]. The exact role of the precuneus in the contrast between the verbal versus the nonverbal conditions with long retention interval requires further investigation.

The short verbal condition showed more brain activity bilaterally around the Sylvian fissure, such as the supramarginal gyrus, which have previously been associated with the *phonological loop *component of *WM *[18,32]. Activity in the supramarginal gyrus has also been related to articulatory rehearsal [18]. For short retention intervals, we were not able to detect any areas that were more active in the non-verbal when compared to the verbal condition (Fig. 4).

This finding suggests that non-verbal *WM *for shorter delay periods depends on different maintenance mechanisms than non-verbal *WM *for longer delay periods. Our study suggests that especially right DLPFC seems to play a crucial role in the maintenance of stimuli in non-verbal *WM*. Since our experiment required the mere maintenance of items without any manipulation process, the results also suggest that DLPFC plays not only a role in manipulation processes [7-9], but also in *WM *maintenance [30]. The differential activity between the verbal and nonverbal conditions (Fig. 4) supports the idea of a dissociation between the left and right hemispheres for verbal and non-verbal *WM*, respectively. Our results are in line with the findings that point to a dominance of the right hemisphere for non-verbal material [31], and these hemispheric differences appear even more pronounced for long retention intervals. One possible reason for the controversy regarding a possible hemispheric specialization for verbal and nonverbal WM might be related to the different retention intervals used in different studies. In a study of Barch et al. [33], the left DLPFC was active for verbal WM only for long delay periods (8 s) as opposed to a short (1 s) retention interval.

## Conclusion

In conclusion, the present study explored the neural correlates of verbal and non-verbal visual *WM *at different delay lengths. Our findings point to a dissociation between verbal and nonverbal WM processing, with a prominent activation of the left hemisphere in verbal coding and a right prefrontal activation associated with non-verbal coding. A recent study by Ikeda and Osaka [32] explored hemispheric differences in inferior frontal and posterior parietal cortex in the verbal and nonverbal encoding of colour stimuli. Together with our findings, these results point to a dissociation of left and right hemispheric processing for verbal and nonverbal working memory for visual stimuli. Furthermore, our findings give rise to the assumption that even slight differences in memory delay length have a significant effect on associated neural networks.

## Competing interests

The author(s) declare that they have no competing interests.

## Authors' contributions

CR, OB and MWG designed the experiment. CR programmed the paradigm. CR and OB collected experimental data. CR, OB and RMM analyzed behavioural and fMRI data. CR, OB and RMM designed and prepared illustrations. CR, OB, TE, SM and MWG wrote the article.
